# Identifying the priority infection prevention and control gaps contributing to neonatal healthcare-associated infections in low-and middle-income countries: results from a modified Delphi process

**DOI:** 10.29392/001c.21367

**Published:** 2021

**Authors:** Daiva Yee, Hanako Osuka, Jamine Weiss, Jane Kriengkauykiat, Amy Kolwaite, Julia Johnson, Joost Hopman, Susan Coffin, Pavani Ram, Florina Serbanescu, Benjamin Park

**Affiliations:** 1 Centers for Disease Control and Prevention, Atlanta, Georgia, USA; 2 Centers for Disease Control and Prevention, Atlanta, Georgia, USA; Chenega Professional and Technical Services, Chesapeake, Virginia, USA; 3 California Department of Public Health, California, USA; 4 Johns Hopkins University, Baltimore, Maryland, USA; 5 Radboud University Medical Center, Nijmegen, Netherlands; 6 Children’s Hospital of Philadelphia, Philadelphia, Pennsylvania, USA; 7 United States Agency for International Development, Washington, District of Columbia, USA

**Keywords:** patient safety, infection prevention and control, low- and middle-income countries, healthcare-associated infections, neonatal sepsis, delphi process

## Abstract

**Background:**

In low- and middle-income countries (LMIC), neonatal healthcare-associated infections (HAI) are associated with increased morbidity, mortality, hospital stay, and costs. When resources are limited, addressing HAI through infection prevention and control (IPC) requires prioritizing interventions to maximize impact. However, little is known about the gaps in LMIC that contribute most to HAI.

**Methods:**

A literature review was conducted to identify the leading IPC gaps contributing to neonatal HAIs in intensive care units and specialty care wards in LMIC. Additionally, a panel of 21 global experts in neonatology and IPC participated in an in-person modified Delphi process to achieve consensus on the relative importance of these gaps as contributors to HAI.

**Results:**

Thirteen IPC gaps were identified and summarized into four main categories: facility policies such as prioritizing a patient safety culture and maintaining facility capacity, general healthcare worker behaviors such as hand hygiene and proper device insertion and maintenance, specialty healthcare worker behaviors such as cleaning and reprocessing of medical equipment, and infrastructural considerations such as adequate medical equipment and hand hygiene supplies.

**Conclusions:**

Through a modified Delphi process, we identified the leading IPC gaps contributing to neonatal HAIs; this information can assist policymakers, public health officials, researchers, and clinicians to prioritize areas for further study or intervention.

Hospital-onset neonatal sepsis, of which bacteremia is a leading cause, is a rapidly growing cause of neonatal morbidity and mortality, prolonging hospital stays and increasing costs.^1,2^ Hospital-onset neonatal sepsis has an estimated annual global burden of 3.0 million cases and an overall mortality of 11–19%.^3^ Rates of neonatal healthcare-associated infections (HAI) in low- and middle-income countries (LMIC) are 3–20 times higher than high-resource settings.^4,5^ Recent studies from LMIC report a majority of invasive bacterial infections in neonates are caused by multi-drug resistant pathogens, organisms that are usually acquired in the healthcare setting.^6,7^

In LMIC, many infection prevention and control (IPC) programs are not fully resourced and therefore cannot adopt all best practices within all key IPC domains. In these cases, IPC programs would have to prioritize the areas that are felt to be the most important and yield the most impact. Although each healthcare setting is likely to be different, in resource-limited settings it is often challenging to conduct a full individualized assessment and identify context-specific priorities; therefore, identifying IPC gaps that are commonly encountered may help policymakers, public health officials, infection preventionists, researchers, and clinicians prioritizing interventions. We sought expert opinions from neonatologists and IPC experts and conducted a modified Delphi process to understand the most common and relevant IPC gaps in neonatal settings in LMIC. Here, we aim to summarize the process of examining common gaps and discuss its implications for policy and practice.

## METHODS

### LITERATURE REVIEW

A literature review was conducted from December 2018 to March 2019. A combination of search terms was used (Appendix S1 in the Online Supplementary Document) to describe neonatal HAIs, IPC, and LMIC. The search was conducted with the following databases: Medline, Embase, Global Health, CINAHL, and Cochrane Library. Following the initial search, additional articles were found manually using the reference list of identified articles. The articles included were based on the eligibility criteria shown in Table 1.

Information was extracted using a standardized data abstraction form. IPC gaps were identified from outbreak reports, cohort studies, case-control studies and intervention studies. Risk factors not related to IPC practices such as low birth weight, prematurity, or maternal health were documented but not analyzed.

Identified IPC gaps were based on expert-opinion grouped in the following categories: invasive devices, hand hygiene, injection safety, receipt of total parenteral or parenteral nutrition (TPN/PN), environmental cleaning, spacing, built environment, staffing and training, and other.

### MODIFIED DELPHI PROCESS PART 1

A group of eight global subject matter experts in neonatology, IPC, and epidemiology in LMIC were identified (Appendix S2 in the Online Supplementary Document) to review the findings from the literature search and provide input into the list of IPC gaps related to neonatal HAIs in LMIC. The list was narrowed to 39 gaps specific to NICU care and excluded those associated with labor, delivery, and prenatal care such as group B streptococcal (GBS) screening or maternal immunization.

Using a modified Delphi process,^9,10^ the experts then individually categorized the gaps based on perceived contribution towards neonatal HAIs. For this process, the participants were asked to rank the 39 identified gaps as very low, low, medium, high, or very high contribution toward neonatal HAIs. Responses were compiled and converted to a numeric scale, 1–5. The median response was then calculated for each gap and results were shared with the group for additional discussion. This process was repeated two more times and included additional criteria for each round, including the size of affected population, applicability to the level of care, and contribution to possible outbreaks. A total of 21 gaps were ranked high or very high and included in the final list.

### MODIFIED DELPHI PROCESS PART 2

To further prioritize the list of 21 high priority gaps, a second modified Delphi process was conducted in a two-day in-person meeting with an additional 13 subject matter experts, including 11 from LMIC (Appendix S2 in the Online Supplementary Document). Participants were asked to rank the gaps from lowest to highest contribution to neonatal HAIs on a numerical scale from 1 to 21.

For each gap, we calculated the percentage of participants who ranked it in the top five and bottom five scores. We then ranked the gaps based on the difference between those percentages. Positive differences indicated a highly ranked gap and negative differences indicated a low ranked gap. Those ranking lowest were removed and the results were shared with the expert group after each round of voting. Discussions were held after each round to build consensus and develop a final list.

## RESULTS

### LITERATURE REVIEW

The database search returned 3,386 articles; 12 articles were added manually. After the removal of duplicates, 2,325 articles remained. Following an initial title and abstract review, 1,987 articles were excluded using the criteria in Table 1. Of the 338 articles undergoing full-text review, 225 were excluded using the same criteria and 113 were included in the analysis (Appendix S3 in the Online Supplementary Document). Among them, 87 were risk studies (eg., out-break reports, cohort studies, case-control studies) and 26 were intervention studies (Table S1 in the Online Supplementary Document).

A total of 141 gaps were identified from 87 risk studies. Table 2 shows the gaps by category. Gaps in IPC practices related to prevention of device-related HAI were most commonly identified (45%): intravenous lines (n=31, 22%), ventilators (n=30, 21%), and tube feeding or transfusion (n=3, 2%). Inadequate hand hygiene was also frequently cited, appearing in 19 (13%) studies followed by inappropriate injection safety in 11 studies (8%).

### MODIFIED DELPHI PROCESS PART 1

Part 1 of the modified Delphi process was conducted in three rounds of ranking with response rates of 63% (5/8), 63% (5/8), and 88% (7/8), respectively. Some gaps such as ‘patient proximity to toilets, infectious waste bins, and sluice rooms’ were deemed less of a concern than ‘unsafe sterilization and reprocessing of medical devices’ and ‘insertion and maintenance of central venous catheters,’ which were ranked as *very high* contribution to HAI. The group also discussed gaps that could potentially lead to an outbreak in the healthcare unit such as ‘poor adherence to processing or sterilization measures,’ ‘reuse of single-use equipment’, and ‘poor healthcare worker compliance with proper hand hygiene.’

After discussing the results with the expert group, the gaps rated as *very high* or *high* were compiled to create a list of the 21 primary IPC gaps contributing to neonatal HAIs (Appendix S4 in the Online Supplementary Document).

### MODIFIED DELPHI PROCESS PART 2

Part 2 of the modified Delphi process was conducted in-person with the goal of establishing the priority areas within 21 primary IPC gaps identified during Part 1. Two rounds of voting occurred with response rates of 90% (19/21) and 95% (20/21), respectively.

In the initial round of the in-person Delphi process, 67% of participants agreed that low healthcare worker (HCW) hand hygiene compliance and inadequate nurse to neonate ratio were in the top five IPC gaps while 100% of participants put availability of personal protective equipment (PPE) in the bottom five IPC gaps. The other gaps that were commonly placed in the bottom five were sink designs that proliferate biofilm (83%), lack of sinks for handwashing (67%), and inadequate cleaning of medical equipment (50%).

These low-ranking gaps were removed for the final round of the process. In this round, 90% of participants agreed that poor patient safety culture was among the top five IPC gaps along with low HCW hand hygiene compliance (75%) and inadequate nurse-to-neonate ratio (65%). Half of the participants placed limited IPC knowledge and practices among primary caregivers and visitors in the bottom five so it was removed from the list.

Following voting and discussion, the final list of 13 primary gaps contributing to neonatal infections in LMIC was determined (Table 3). These 13 gaps were then categorized as facility policies, general healthcare worker behaviors, specialty healthcare worker behaviors, and infrastructure and consumables. Lack of patient safety culture, in particular, was identified as one of the most important IPC gaps contributing to neonatal HAIs.

## DISCUSSION

To our knowledge, this is the first attempt at identifying the most common, and most important, IPC gaps that contribute to neonatal sepsis in LMIC. Our final list is based on a review of the literature, expert opinion, and experience working in resource-limited neonatal wards. This information may be used by policymakers, public health officials, clinicians, and researchers to prioritize interventions.

The group agreed that poor patient safety culture was one of the most important contributors to weak IPC in healthcare facilities. Improving patient safety culture, defined as the product of individual and group beliefs, values, attitudes, perceptions, competencies, and patterns of behavior that determine the organization’s commitment to quality and patient safety,^11^ has been demonstrated to lower HAI rates.^12^ Several studies have demonstrated that interventions targeting patient safety culture, including comprehensive unit-based safety programs (CUSP), can be a highly important intervention to prevent HAI.^13–16^ CUSP is a method that includes both technical and socioadaptive aspects, and it can help clinical teams make care safer by combining improved teamwork, clinical best practices, and the science of safety. This approach has been attempted in LMIC with some success.^17^

We identified some important gaps that IPC specialists often do not have the mandate to address in LMIC. For example, improper sterile compounding and use of multi-dose vials or solutions were identified as critically important risk factors for infection; however, these tasks typically are conducted by clinical pharmacists or clinical nurses. Likewise, the group also identified inadequate environmental cleaning and reprocessing of equipment; these tasks are often carried out by contracted cleaning staff. Establishing linkages with IPC to these services and professions can be critical to address these deficiencies.

The group identified lack of human resources or space (e.g., inadequate nurse-to-neonate ratio; multiple neonates in a single incubator/warmer; exceeding facility capacity) as being major contributors to poor IPC. Understaffing and overcrowding have been well-described in the literature as risk factors for HAIs.^18–20^ While it is certainly not surprising that these factors, which are very resource-intensive to rectify, were seen as vital contributors to risk, it was recognized that many of these problems stem from the same common underlying issue of overcrowding and a lack of sufficient special care for small and sick newborns.^21^ Advocating for “right-sized” neonatal wards or admissions policies, where the number of patients do not exceed the capacity of the facility, is one critical policy that could make a substantial difference in addressing these gaps. This implies a potential need for government buy-in for these policies to be put in place. Recognition that these gaps require policy solutions has been increasingly evident. In South Africa, for example, a framework for a nationally endorsed HAI prevention strategy features the need for IPC policy and infrastructure development.^22^

There are several limitations to our process. First, the data from our literature search was limited by the detail and quality of the original articles. Some articles did not provide detailed information about each IPC gap; for example, if the device was identified as an IPC gap, it is also possible that the facility also had inappropriate hand hygiene practice or a lack of safety culture. Similarly, it was sometimes difficult to assess whether gaps were identified through conjecture or epidemiological or biological evidence. Second, since the first rounds of voting were facilitated via email, there were low response rates in part 1 of the Delphi process contributing to a small sample size. Third, although there was good consensus among participants, these data are based on the collective experience of the group and may not be applicable to all similar settings.

## CONCLUSIONS

Using a modified Delphi approach, we developed a list of some of the most important IPC gaps to address to prevent neonatal HAI in LMIC. Although additional epidemiological research is needed, these data can be used immediately to approach IPC improvements in neonatal settings to reduce risk of infection and death among this vulnerable population. Our study included experts from various countries and backgrounds and thus this study does not represent any one specific country. Since the epidemiological situation and resources vary by country, conducting a similar process with neonatal and IPC experts in specific country settings may be recommended for a more tailored approach. Future work should also include systematic assessments of neonatal settings to improve our understanding of baseline IPC conditions, in addition to published implementation science studies highlighting the challenges and solutions for reducing neonatal infection in LMIC.

## Supplementary Material

Supplementary Material

## Figures and Tables

**Table 1:** Eligibility criteria for literature review

Inclusion Criteria:

• NICU setting • IPC gaps contributing to neonatal HAI • IPC interventions to reduce infection • Original articles from low- and middle-income countries (based on 2018 World Bank definition)^8^ • Full-text available

Exclusion Criteria:

• Treatment or prophylaxis drug use • Non-healthcare-associated infections • Non-neonatal populations • Non-healthcare settings • Non-English languages • Commentary, conference abstracts

NICU: neonatal intensive care unit, IPC: infection prevention and control, HAI: healthcare-associated infection

**Table 2:** Identified IPC gaps from risk studies (n=87)

IPC gaps by category	Number	(%)

Device	64	45
• Venous line (CV, PICC, other)	31	22
• Ventilation*	30	21
• Other device**	3	2
Hand hygiene	19	13
Injection safety	11	8
TPN/PN	10	7
Environmental cleaning	8	6
Spacing	5	4
Built environment	2	1
Staffing and training	1	1
Other	21	15
**Total**	**141**	**100**

IPC: infection prevention and control, CVC: central venous catheter, PICC: peripherally inserted central catheter, TPN: total parenteral nutrition

* Ventilation includes: mechanical ventilation, prolonged ventilation, intubation, and reintubation

** Other devices include: tube feeding and transfusion

**Table 3:** Final list of IPC gaps most contributing to neonatal HAI in LMIC as determined by the modified Delphi process

Facility policies:

• Poor patient safety culture • Inadequate nurse to neonate ratio • Multiple neonates in a single patient incubator or warmer • Exceeds facility capacity

General healthcare worker behaviors:

• Low HCW (and caregiver) hand hygiene compliance • Improper aseptic technique for device insertion • Poor adherence to device and IV maintenance, care, and administration

Specialty healthcare worker behaviors:

• Inadequate cleaning of non-critical items in contact with skin • Inadequate sterilization or reprocessing of multi-use, semi-critical and critical items • Improper sterile compounding of medication or fluids and improper multi-dose vial or solution use

Infrastructure and consumables:

• Reuse of single use semi-critical and critical items • Lack of alcohol-based hand rub at point of care • Lack of running water, sink, soap and paper towels on ward

HAI: healthcare-associated infection, HCW: healthcare worker, IV: intravenous, LMIC: low- and middle-income countries
