# Supplementary material for: Identifying the priority infection prevention and control gaps contributing to neonatal healthcare-associated infections in low-and middle-income countries: results from a modified Delphi process
Source: J Glob Health Rep. Author manuscript; Available in PMC 2023 May 11. (PMC10174223; doi:10.29392/001c.21367)
Supplement: Supplementary Material [file NIHMS1842242-supplement-Supplementary_Material.docx]

**Online Supplementary Documents**

**Appendix S1: Literature review search query and strategy**

**Search Query:** Primary infection control risk factors contributing to neonatal nosocomial infection in low-resource neonatal wards. Keywords: neonatal infection; neonatal sepsis; infection control and prevention; low-resource settings; low- and middle-income countries; nosocomial infection; healthcare-associated infection; hospital neonatal wards

**Search Strategy:**

| **Database** | **Strategy** | **Run Date** | **Records** |
| --- | --- | --- | --- |
| **Medline**  **(OVID)**  **1946-** | neonatal sepsis OR neonatal sepses OR (neonatal ADJ5 infection*) OR (newborn* ADJ5 infection*) OR (infant* ADJ5 infection*) OR (neonatal ADJ5 pneumonia) OR (newborn* ADJ5 pneumonia) OR (infant* ADJ5 pneumonia)  AND  nosocomial OR healthcare-associated OR hospital ward* OR hospital unit* OR Hospital-acquired OR healthcare-acquired OR health-care acquired OR health-care associated OR cross infection* OR neonatal ward* OR neonatal intensive care OR neonatal unit* OR NICU OR hospital infection* OR device-associated OR catheter* OR central line OR ventilator* OR surgical site* OR hospital diarrhea  AND  Infection control OR infection prevention OR (prevention ADJ5 control) OR stewardship OR pc.fs OR risk* OR cause* OR transmi* OR contaminat* OR Hand hygiene OR Hand disinfection OR Hand washing OR Handwashing OR Infection control OR precaution* OR surveillance OR equipment OR decontamination OR cleaning OR disinfection OR steriliz* OR isolation OR education OR training OR learning OR knowledge OR competence OR bundle OR checklist* OR intervention* OR audit* OR management OR guideline* OR Innovation OR quality Improvement* OR quality control* OR practic* OR routine OR procedure* OR requirement* OR policy OR policies OR strateg* OR care pathway*  AND  (  low-resource* OR middle-income countr* OR LMIC* OR developing countr* OR third world OR transitional countr* OR ((underserved OR under-served OR deprived OR poor*) ADJ5 (countr* OR nation? OR population? OR world)) OR ((developing or less* developed or under developed or underdeveloped or middle income or low* income) ADJ5 (economy or economies))  OR  (Afghanistan or Albania or Algeria or Angola or Antigua or Barbuda or Argentina or Armenia or Armenian or Aruba or Azerbaijan or Bahrain or Bangladesh or Barbados or Benin or Byelarus or Byelorussian or Belarus or Belorussian or Belorussia or Belize or Bhutan or Bolivia or Bosnia or Herzegovina or Hercegovina or Botswana or Brasil or Brazil or Bulgaria or Burkina Faso or Burkina Fasso or Upper Volta or Burundi or Urundi or Cambodia or Khmer Republic or Kampuchea or Cameroon or Cameroons or Cameron or Camerons or Cape Verde or Central African Republic or Chad or Chile or China or Colombia or Comoros or Comoro Islands or Comores or Mayotte or Congo or Zaire or Costa Rica or Cote d'Ivoire or Ivory Coast or Croatia or Cuba or Cyprus or Czechoslovakia or Czech Republic or Slovakia or Slovak Republic or Djibouti or French Somaliland or Dominica or Dominican Republic or East Timor or East Timur or Timor Leste or Ecuador or Egypt or United Arab Republic or El Salvador or Eritrea or Estonia or Ethiopia or Fiji or Gabon or Gabonese Republic or Gambia or Gaza or Georgia Republic or Georgian Republic or Ghana or Gold Coast or Greece or Grenada or Guatemala or Guinea or Guam or Guiana or Guyana or Haiti or Honduras or Hungary or India or Maldives or Indonesia or Iran or Iraq or Isle of Man or Jamaica or Jordan or Kazakhstan or Kazakh or Kenya or Kiribati or Korea or Kosovo or Kyrgyzstan or Kirghizia or Kyrgyz Republic or Kirghiz or Kirgizstan or Lao PDR or Laos or Latvia or Lebanon or Lesotho or Basutoland or Liberia or Libya or Lithuania or Macedonia or Madagascar or Malagasy Republic or Malaysia or Malaya or Malay or Sabah or Sarawak or Malawi or Nyasaland or Mali or Malta or Marshall Islands or Mauritania or Mauritius or Agalega Islands or Mexico or Micronesia or Middle East or Moldova or Moldovia or Moldovian or Mongolia or Montenegro or Morocco or Ifni or Mozambique or Myanmar or Myanma or Burma or Namibia or Nepal or Netherlands Antilles or New Caledonia or Nicaragua or Niger or Nigeria or Northern Mariana Islands or Oman or Muscat or Pakistan or Palau or Palestine or Panama or Paraguay or Peru or Philippines or Philipines or Phillipines or Phillippines or Poland or Portugal or Puerto Rico or Romania or Rumania or Roumania or Russia or Russian or Rwanda or Ruanda or Saint Kitts or St Kitts or Nevis or Saint Lucia or St Lucia or Saint Vincent or St Vincent or Grenadines or Samoa or Samoan Islands or Navigator Island or Navigator Islands or Sao Tome or Saudi Arabia or Senegal or Serbia or Montenegro or Seychelles or Sierra Leone or Slovenia or Sri Lanka or Ceylon or Solomon Islands or Somalia or South Africa or Sudan or Suriname or Surinam or Swaziland or Syria or Tajikistan or Tadzhikistan or Tadjikistan or Tadzhik or Tanzania or Thailand or Togo or Togolese Republic or Tonga or Trinidad or Tobago or Tunisia or Turkey or Turkmenistan or Turkmen or Uganda or Ukraine or Uruguay or USSR or Soviet Union or Union of Soviet Socialist Republics or Uzbekistan or Uzbek or Vanuatu or New Hebrides or Venezuela or Vietnam or Viet Nam or West Bank or Yemen or Yugoslavia or Zambia or Zimbabwe or Rhodesia).hw,kf,ti,ab,cp.  )  Abstracts Available | 12/17/2018 | 768 |
| **Embase**  **(OVID)**  **1947-** | neonatal sepsis OR neonatal sepses OR (neonatal ADJ5 infection*) OR (newborn* ADJ5 infection*) OR (infant* ADJ5 infection*) OR (neonatal ADJ5 pneumonia) OR (newborn* ADJ5 pneumonia) OR (infant* ADJ5 pneumonia)  AND  nosocomial OR healthcare-associated OR hospital ward* OR hospital unit* OR Hospital-acquired OR healthcare-acquired OR health-care acquired OR health-care associated OR cross infection* OR neonatal ward* OR neonatal intensive care OR neonatal unit* OR NICU OR hospital infection* OR device-associated OR catheter* OR central line OR ventilator* OR surgical site* OR hospital diarrhea  AND  Infection control OR infection prevention OR (prevention ADJ5 control) OR stewardship OR pc.fs OR risk* OR cause* OR transmi* OR contaminat* OR Hand hygiene OR Hand disinfection OR Hand washing OR Handwashing OR Infection control OR precaution* OR surveillance OR equipment OR decontamination OR cleaning OR disinfection OR steriliz* OR isolation OR education OR training OR learning OR knowledge OR competence OR bundle OR checklist* OR intervention* OR audit* OR management OR guideline* OR Innovation OR quality Improvement* OR quality control* OR practic* OR routine OR procedure* OR requirement* OR policy OR policies OR strateg* OR care pathway*  AND  (  low-resource* OR middle-income countr* OR LMIC* OR developing countr* OR third world OR transitional countr* OR ((underserved OR under-served OR deprived OR poor*) ADJ5 (countr* OR nation? OR population? OR world)) OR ((developing or less* developed or under developed or underdeveloped or middle income or low* income) ADJ5 (economy or economies))  OR  (Afghanistan or Albania or Algeria or Angola or Antigua or Barbuda or Argentina or Armenia or Armenian or Aruba or Azerbaijan or Bahrain or Bangladesh or Barbados or Benin or Byelarus or Byelorussian or Belarus or Belorussian or Belorussia or Belize or Bhutan or Bolivia or Bosnia or Herzegovina or Hercegovina or Botswana or Brasil or Brazil or Bulgaria or Burkina Faso or Burkina Fasso or Upper Volta or Burundi or Urundi or Cambodia or Khmer Republic or Kampuchea or Cameroon or Cameroons or Cameron or Camerons or Cape Verde or Central African Republic or Chad or Chile or China or Colombia or Comoros or Comoro Islands or Comores or Mayotte or Congo or Zaire or Costa Rica or Cote d'Ivoire or Ivory Coast or Croatia or Cuba or Cyprus or Czechoslovakia or Czech Republic or Slovakia or Slovak Republic or Djibouti or French Somaliland or Dominica or Dominican Republic or East Timor or East Timur or Timor Leste or Ecuador or Egypt or United Arab Republic or El Salvador or Eritrea or Estonia or Ethiopia or Fiji or Gabon or Gabonese Republic or Gambia or Gaza or Georgia Republic or Georgian Republic or Ghana or Gold Coast or Greece or Grenada or Guatemala or Guinea or Guam or Guiana or Guyana or Haiti or Honduras or Hungary or India or Maldives or Indonesia or Iran or Iraq or Isle of Man or Jamaica or Jordan or Kazakhstan or Kazakh or Kenya or Kiribati or Korea or Kosovo or Kyrgyzstan or Kirghizia or Kyrgyz Republic or Kirghiz or Kirgizstan or Lao PDR or Laos or Latvia or Lebanon or Lesotho or Basutoland or Liberia or Libya or Lithuania or Macedonia or Madagascar or Malagasy Republic or Malaysia or Malaya or Malay or Sabah or Sarawak or Malawi or Nyasaland or Mali or Malta or Marshall Islands or Mauritania or Mauritius or Agalega Islands or Mexico or Micronesia or Middle East or Moldova or Moldovia or Moldovian or Mongolia or Montenegro or Morocco or Ifni or Mozambique or Myanmar or Myanma or Burma or Namibia or Nepal or Netherlands Antilles or New Caledonia or Nicaragua or Niger or Nigeria or Northern Mariana Islands or Oman or Muscat or Pakistan or Palau or Palestine or Panama or Paraguay or Peru or Philippines or Philipines or Phillipines or Phillippines or Poland or Portugal or Puerto Rico or Romania or Rumania or Roumania or Russia or Russian or Rwanda or Ruanda or Saint Kitts or St Kitts or Nevis or Saint Lucia or St Lucia or Saint Vincent or St Vincent or Grenadines or Samoa or Samoan Islands or Navigator Island or Navigator Islands or Sao Tome or Saudi Arabia or Senegal or Serbia or Montenegro or Seychelles or Sierra Leone or Slovenia or Sri Lanka or Ceylon or Solomon Islands or Somalia or South Africa or Sudan or Suriname or Surinam or Swaziland or Syria or Tajikistan or Tadzhikistan or Tadjikistan or Tadzhik or Tanzania or Thailand or Togo or Togolese Republic or Tonga or Trinidad or Tobago or Tunisia or Turkey or Turkmenistan or Turkmen or Uganda or Ukraine or Uruguay or USSR or Soviet Union or Union of Soviet Socialist Republics or Uzbekistan or Uzbek or Vanuatu or New Hebrides or Venezuela or Vietnam or Viet Nam or West Bank or Yemen or Yugoslavia or Zambia or Zimbabwe or Rhodesia).hw,kf,ti,ab,cp.  )  Abstracts Available | 12/17/2018 | 1473  -526  duplicates  =947  unique items |
| **Global Health**  **(OVID)**  **1967-** | neonatal sepsis OR neonatal sepses OR (neonatal ADJ5 infection*) OR (newborn* ADJ5 infection*) OR (infant* ADJ5 infection*) OR (neonatal ADJ5 pneumonia) OR (newborn* ADJ5 pneumonia) OR (infant* ADJ5 pneumonia)  AND  nosocomial OR healthcare-associated OR hospital ward* OR hospital unit* OR Hospital-acquired OR healthcare-acquired OR health-care acquired OR health-care associated OR cross infection* OR neonatal ward* OR neonatal intensive care OR neonatal unit* OR NICU OR hospital infection* OR device-associated OR catheter* OR central line OR ventilator* OR surgical site* OR hospital diarrhea  AND  Infection control OR infection prevention OR (prevention ADJ5 control) OR stewardship OR risk* OR cause* OR transmi* OR contaminat* OR Hand hygiene OR Hand disinfection OR Hand washing OR Handwashing OR Infection control OR precaution* OR surveillance OR equipment OR decontamination OR cleaning OR disinfection OR steriliz* OR isolation OR education OR training OR learning OR knowledge OR competence OR bundle OR checklist* OR intervention* OR audit* OR management OR guideline* OR Innovation OR quality Improvement* OR quality control* OR practic* OR routine OR procedure* OR requirement* OR policy OR policies OR strateg* OR care pathway*  AND  (  low-resource* OR middle-income countr* OR LMIC* OR developing countr* OR third world OR transitional countr* OR ((underserved OR under-served OR deprived OR poor*) ADJ5 (countr* OR nation? OR population? OR world)) OR ((developing or less* developed or under developed or underdeveloped or middle income or low* income) ADJ5 (economy or economies))  OR  (Afghanistan or Albania or Algeria or Angola or Antigua or Barbuda or Argentina or Armenia or Armenian or Aruba or Azerbaijan or Bahrain or Bangladesh or Barbados or Benin or Byelarus or Byelorussian or Belarus or Belorussian or Belorussia or Belize or Bhutan or Bolivia or Bosnia or Herzegovina or Hercegovina or Botswana or Brasil or Brazil or Bulgaria or Burkina Faso or Burkina Fasso or Upper Volta or Burundi or Urundi or Cambodia or Khmer Republic or Kampuchea or Cameroon or Cameroons or Cameron or Camerons or Cape Verde or Central African Republic or Chad or Chile or China or Colombia or Comoros or Comoro Islands or Comores or Mayotte or Congo or Zaire or Costa Rica or Cote d'Ivoire or Ivory Coast or Croatia or Cuba or Cyprus or Czechoslovakia or Czech Republic or Slovakia or Slovak Republic or Djibouti or French Somaliland or Dominica or Dominican Republic or East Timor or East Timur or Timor Leste or Ecuador or Egypt or United Arab Republic or El Salvador or Eritrea or Estonia or Ethiopia or Fiji or Gabon or Gabonese Republic or Gambia or Gaza or Georgia Republic or Georgian Republic or Ghana or Gold Coast or Greece or Grenada or Guatemala or Guinea or Guam or Guiana or Guyana or Haiti or Honduras or Hungary or India or Maldives or Indonesia or Iran or Iraq or Isle of Man or Jamaica or Jordan or Kazakhstan or Kazakh or Kenya or Kiribati or Korea or Kosovo or Kyrgyzstan or Kirghizia or Kyrgyz Republic or Kirghiz or Kirgizstan or Lao PDR or Laos or Latvia or Lebanon or Lesotho or Basutoland or Liberia or Libya or Lithuania or Macedonia or Madagascar or Malagasy Republic or Malaysia or Malaya or Malay or Sabah or Sarawak or Malawi or Nyasaland or Mali or Malta or Marshall Islands or Mauritania or Mauritius or Agalega Islands or Mexico or Micronesia or Middle East or Moldova or Moldovia or Moldovian or Mongolia or Montenegro or Morocco or Ifni or Mozambique or Myanmar or Myanma or Burma or Namibia or Nepal or Netherlands Antilles or New Caledonia or Nicaragua or Niger or Nigeria or Northern Mariana Islands or Oman or Muscat or Pakistan or Palau or Palestine or Panama or Paraguay or Peru or Philippines or Philipines or Phillipines or Phillippines or Poland or Portugal or Puerto Rico or Romania or Rumania or Roumania or Russia or Russian or Rwanda or Ruanda or Saint Kitts or St Kitts or Nevis or Saint Lucia or St Lucia or Saint Vincent or St Vincent or Grenadines or Samoa or Samoan Islands or Navigator Island or Navigator Islands or Sao Tome or Saudi Arabia or Senegal or Serbia or Montenegro or Seychelles or Sierra Leone or Slovenia or Sri Lanka or Ceylon or Solomon Islands or Somalia or South Africa or Sudan or Suriname or Surinam or Swaziland or Syria or Tajikistan or Tadzhikistan or Tadjikistan or Tadzhik or Tanzania or Thailand or Togo or Togolese Republic or Tonga or Trinidad or Tobago or Tunisia or Turkey or Turkmenistan or Turkmen or Uganda or Ukraine or Uruguay or USSR or Soviet Union or Union of Soviet Socialist Republics or Uzbekistan or Uzbek or Vanuatu or New Hebrides or Venezuela or Vietnam or Viet Nam or West Bank or Yemen or Yugoslavia or Zambia or Zimbabwe or Rhodesia).sh,ti,ab.  )  Abstracts Available | 12/17/2018 | 915  -385  duplicates  =530  unique items |
| **CINAHL**  **(Ebsco)** | “neonatal sepsis” OR “neonatal sepses” OR (neonatal N5 infection*) OR (newborn* N5 infection*) OR (infant* N5 infection*) OR (neonatal N5 pneumonia) OR (newborn* N5 pneumonia) OR (infant* N5 pneumonia)  AND  nosocomial OR healthcare-associated OR “hospital ward*” OR “hospital unit*” OR Hospital-acquired OR healthcare-acquired OR “health-care acquired” OR “health-care associated” OR “cross infection*” OR “neonatal ward*” OR “neonatal intensive care” OR “neonatal unit*” OR NICU OR “hospital infection*” OR device-associated OR catheter* OR “central line” OR ventilator* OR “surgical site*” OR “hospital diarrhea”  AND  “Infection control” OR “infection prevention” OR (prevention N5 control) OR stewardship OR risk* OR cause* OR transmi* OR contaminat* OR “Hand hygiene” OR “Hand disinfection” OR “Hand washing” OR Handwashing OR precaution* OR surveillance OR equipment OR decontamination OR cleaning OR disinfection OR steriliz* OR isolation OR education OR training OR learning OR knowledge OR competence OR bundle OR checklist* OR intervention* OR audit* OR management OR guideline* OR innovation OR “quality Improvement*” OR “quality control*” OR practic* OR routine OR procedure* OR requirement* OR policy OR policies OR strateg* OR “care pathway*”  AND  (  “low-resource*” OR “middle-income countr*” OR LMIC* OR “developing countr*” OR “third world” OR “transitional countr*” OR ((underserved OR under-served OR deprived OR poor*) N5 (countr* OR nation? OR population? OR world)) OR ((developing or less* developed or “under developed” or underdeveloped or “middle income” or “low* income”) N5 (economy or economies))  OR  (Afghanistan or Albania or Algeria or Angola or Antigua or Barbuda or Argentina or Armenia or Armenian or Aruba or Azerbaijan or Bahrain or Bangladesh or Barbados or Benin or Byelarus or Byelorussian or Belarus or Belorussian or Belorussia or Belize or Bhutan or Bolivia or Bosnia or Herzegovina or Hercegovina or Botswana or Brasil or Brazil or Bulgaria or “Burkina Faso” or “Burkina Fasso” or “Upper Volta” or Burundi or Urundi or Cambodia or “Khmer Republic” or Kampuchea or Cameroon or Cameroons or Cameron or Camerons or “Cape Verde” or “Central African Republic” or Chad or Chile or China or Colombia or Comoros or “Comoro Islands” or Comores or Mayotte or Congo or Zaire or Costa Rica or “Cote d'Ivoire” or “Ivory Coast” or Croatia or Cuba or Cyprus or Czechoslovakia or “Czech Republic” or Slovakia or “Slovak Republic” or Djibouti or “French Somaliland” or Dominica or “Dominican Republic” or “East Timor” or “East Timur” or “Timor Leste” or Ecuador or Egypt or “United Arab Republic” or “El Salvador” or Eritrea or Estonia or Ethiopia or Fiji or Gabon or “Gabonese Republic” or Gambia or Gaza or “Georgia Republic” or “Georgian Republic” or Ghana or “Gold Coast” or Greece or Grenada or Guatemala or Guinea or Guam or Guiana or Guyana or Haiti or Honduras or Hungary or India or Maldives or Indonesia or Iran or Iraq or “Isle of Man” or Jamaica or Jordan or Kazakhstan or Kazakh or Kenya or Kiribati or Korea or Kosovo or Kyrgyzstan or Kirghizia or “Kyrgyz Republic” or Kirghiz or Kirgizstan or “Lao PDR” or Laos or Latvia or Lebanon or Lesotho or Basutoland or Liberia or Libya or Lithuania or Macedonia or Madagascar or “Malagasy Republic” or Malaysia or Malaya or Malay or Sabah or Sarawak or Malawi or Nyasaland or Mali or Malta or “Marshall Islands” or Mauritania or Mauritius or “Agalega Islands” or Mexico or Micronesia or Middle East or Moldova or Moldovia or Moldovian or Mongolia or Montenegro or Morocco or Ifni or Mozambique or Myanmar or Myanma or Burma or Namibia or Nepal or “Netherlands Antilles” or “New Caledonia” or Nicaragua or Niger or Nigeria or “Northern Mariana Islands” or Oman or Muscat or Pakistan or Palau or Palestine or Panama or Paraguay or Peru or Philippines or Philipines or Phillipines or Phillippines or Poland or Portugal or “Puerto Rico” or Romania or Rumania or Roumania or Russia or Russian or Rwanda or Ruanda or “Saint Kitts” or “St Kitts” or Nevis or “Saint Lucia” or “St Lucia” or “Saint Vincent” or “St Vincent” or Grenadines or Samoa or “Samoan Islands” or “Navigator Island” or “Navigator Islands” or “Sao Tome” or “Saudi Arabia” or Senegal or Serbia or Montenegro or Seychelles or “Sierra Leone” or Slovenia or “Sri Lanka” or Ceylon or “Solomon Islands” or Somalia or “South Africa” or Sudan or Suriname or Surinam or Swaziland or Syria or Tajikistan or Tadzhikistan or Tadjikistan or Tadzhik or Tanzania or Thailand or Togo or “Togolese Republic” or Tonga or Trinidad or Tobago or Tunisia or Turkey or Turkmenistan or Turkmen or Uganda or Ukraine or Uruguay or USSR or “Soviet Union” or “Union of Soviet Socialist Republics” or Uzbekistan or Uzbek or Vanuatu or “New Hebrides” or Venezuela or Vietnam or “Viet Nam” or “West Bank” or Yemen or Yugoslavia or Zambia or Zimbabwe or Rhodesia)  )  Abstracts Available | 12/17/2018 | 179  -133  duplicates  =46  unique items |
| **Cochrane Library** | (“neonatal sepsis” OR “neonatal sepses” OR (neonatal NEAR/5 infection*) OR (newborn* NEAR/5 infection*) OR (infant* NEAR/5 infection*) OR (neonatal NEAR/5 pneumonia) OR (newborn* NEAR/5 pneumonia) OR (infant* NEAR/5 pneumonia)):ti,ab  AND  (nosocomial OR healthcare-associated OR “hospital ward*” OR “hospital unit*” OR Hospital-acquired OR healthcare-acquired OR “health-care acquired” OR “health-care associated” OR “cross infection*” OR “neonatal ward*” OR “neonatal intensive care” OR “neonatal unit*” OR NICU OR “hospital infection*” OR device-associated OR catheter* OR “central line” OR ventilator* OR “surgical site*” OR “hospital diarrhea”):ti,ab  AND  (“Infection control” OR “infection prevention” OR (prevention NEAR/5 control) OR stewardship OR risk* OR cause* OR transmi* OR contaminat* OR “Hand hygiene” OR “Hand disinfection” OR “Hand washing” OR Handwashing OR precaution* OR surveillance OR equipment OR decontamination OR cleaning OR disinfection OR steriliz* OR isolation OR education OR training OR learning OR knowledge OR competence OR bundle OR checklist* OR intervention* OR audit* OR management OR guideline* OR innovation OR “quality improvement*” OR “quality control*” OR practic* OR routine OR procedure* OR requirement* OR policy OR policies OR strateg* OR “care pathway*”):ti,ab  AND  (  (“low-resource*” OR “middle-income countr*” OR LMIC* OR “developing countr*” OR “third world” OR “transitional countr*” OR ((underserved OR under-served OR deprived OR poor*) NEAR/5 (countr* OR nation? OR population? OR world)) OR ((developing or less* developed or “under developed” or underdeveloped or “middle income” or “low* income”) NEAR/5 (economy or economies))):ti,ab  OR  (Afghanistan or Albania or Algeria or Angola or Antigua or Barbuda or Argentina or Armenia or Armenian or Aruba or Azerbaijan or Bahrain or Bangladesh or Barbados or Benin or Byelarus or Byelorussian or Belarus or Belorussian or Belorussia or Belize or Bhutan or Bolivia or Bosnia or Herzegovina or Hercegovina or Botswana or Brasil or Brazil or Bulgaria or “Burkina Faso” or “Burkina Fasso” or “Upper Volta” or Burundi or Urundi or Cambodia or “Khmer Republic” or Kampuchea or Cameroon or Cameroons or Cameron or Camerons or “Cape Verde” or “Central African Republic” or Chad or Chile or China or Colombia or Comoros or “Comoro Islands” or Comores or Mayotte or Congo or Zaire or Costa Rica or “Cote d'Ivoire” or “Ivory Coast” or Croatia or Cuba or Cyprus or Czechoslovakia or “Czech Republic” or Slovakia or “Slovak Republic” or Djibouti or “French Somaliland” or Dominica or “Dominican Republic” or “East Timor” or “East Timur” or “Timor Leste” or Ecuador or Egypt or “United Arab Republic” or “El Salvador” or Eritrea or Estonia or Ethiopia or Fiji or Gabon or “Gabonese Republic” or Gambia or Gaza or “Georgia Republic” or “Georgian Republic” or Ghana or “Gold Coast” or Greece or Grenada or Guatemala or Guinea or Guam or Guiana or Guyana or Haiti or Honduras or Hungary or India or Maldives or Indonesia or Iran or Iraq or “Isle of Man” or Jamaica or Jordan or Kazakhstan or Kazakh or Kenya or Kiribati or Korea or Kosovo or Kyrgyzstan or Kirghizia or “Kyrgyz Republic” or Kirghiz or Kirgizstan or “Lao PDR” or Laos or Latvia or Lebanon or Lesotho or Basutoland or Liberia or Libya or Lithuania or Macedonia or Madagascar or “Malagasy Republic” or Malaysia or Malaya or Malay or Sabah or Sarawak or Malawi or Nyasaland or Mali or Malta or “Marshall Islands” or Mauritania or Mauritius or “Agalega Islands” or Mexico or Micronesia or Middle East or Moldova or Moldovia or Moldovian or Mongolia or Montenegro or Morocco or Ifni or Mozambique or Myanmar or Myanma or Burma or Namibia or Nepal or “Netherlands Antilles” or “New Caledonia” or Nicaragua or Niger or Nigeria or “Northern Mariana Islands” or Oman or Muscat or Pakistan or Palau or Palestine or Panama or Paraguay or Peru or Philippines or Philipines or Phillipines or Phillippines or Poland or Portugal or “Puerto Rico” or Romania or Rumania or Roumania or Russia or Russian or Rwanda or Ruanda or “Saint Kitts” or “St Kitts” or Nevis or “Saint Lucia” or “St Lucia” or “Saint Vincent” or “St Vincent” or Grenadines or Samoa or “Samoan Islands” or “Navigator Island” or “Navigator Islands” or “Sao Tome” or “Saudi Arabia” or Senegal or Serbia or Montenegro or Seychelles or “Sierra Leone” or Slovenia or “Sri Lanka” or Ceylon or “Solomon Islands” or Somalia or “South Africa” or Sudan or Suriname or Surinam or Swaziland or Syria or Tajikistan or Tadzhikistan or Tadjikistan or Tadzhik or Tanzania or Thailand or Togo or “Togolese Republic” or Tonga or Trinidad or Tobago or Tunisia or Turkey or Turkmenistan or Turkmen or Uganda or Ukraine or Uruguay or USSR or “Soviet Union” or “Union of Soviet Socialist Republics” or Uzbekistan or Uzbek or Vanuatu or “New Hebrides” or Venezuela or Vietnam or “Viet Nam” or “West Bank” or Yemen or Yugoslavia or Zambia or Zimbabwe or Rhodesia):ti,ab  )  Abstracts Available | 12/17/2018 | 39  -29  duplicates  =10  unique items |

**Appendix S2:** **List of participants**

| **Name** | **Organization** | **Country** |
| --- | --- | --- |
| Modified Delphi Part 1 Participants | | |
| Susan Coffin | Children’s Hospital of Philadelphia | United States |
| Joost Hopman | Radboud University Medical Center | Netherlands |
| Julia Johnson | Johns Hopkins University | United States |
| Benjamin Park | U.S. Centers for Disease Control and Prevention | United States |
| Pavani Ram | United States Agency for International Development | United States |
| Florina Serbanescu | U.S. Centers for Disease Control and Prevention | United States |
| Rachel Smith | U.S. Centers for Disease Control and Prevention | United States |
| Matthew Westercamp | U.S. Centers for Disease Control and Prevention | United States |
| Modified Delphi Part 2 Participants | | |
| Susan Coffin | Children’s Hospital of Philadelphia | United States |
| Melanie Curless | Johns Hopkins University | Malaysia |
| Angela Dramowski | Stellenbosch University | South Africa |
| Queen Dube | University of Malawi | Malawi |
| Danielle Ehret | University of Vermont Children’s Hospital | United States |
| Chika Christabel Enweronu-Laryea | University of Ghana School of Medicine | Ghana |
| Amy Ginsburg | Save the Children | United States |
| Joost Hopman | Radboud University Medical Center | Netherlands |
| Julia Johnson | Johns Hopkins University | United States |
| Amy Kolwaite | U.S. Centers for Disease Control and Prevention | United States |
| Michuki Maina | KEMRI Wellcome Trust | Kenya |
| Jeevasankar Mari | All India Institute of Medical Sciences | India |
| Lawrence Mwananyanda | Right to Care | Zambia |
| Tochi Okwor | Nigeria Center for Disease Control | Nigeria |
| Benjamin Park | U.S. Centers for Disease Control and Prevention | United States |
| Pavani Ram | United States Agency for International Development | United States |
| Florina Serbanescu | U.S. Centers for Disease Control and Prevention | United States |
| Nalini Singh | The George Washington University | United States |
| David Tsibadze | New Hospitals | Georgia |
| Sithembiso Velaphi | University of Witwatersrand | South Africa |
| Peter Waiswa | Makerere University School of Public Health | Uganda |

**Appendix S3: Flow chart of literature review**

**Identification**

Records identified in Medline, Embase, Global Health, CINAHL, Cochrane Library
(n = 3,386)

Records identified manually
(n = 12)

**Included**

**Eligibility**

Full-text articles assessed for eligibility
(n = 338)

Records screened
(n = 2,325)

Records excluded
(n = 1,987)

Records after duplicates removed
(n = 2,325)

**Screening**

**Eligibility**

Full-text articles excluded if did not meet search criteria
(n = 225)

**Included**

Studies included in analysis
(n = 113)

**Appendix S4: 21 primary IPC gaps contributing to neonatal HAIs in the modified Delphi process part 1**

| **Gap Category** | **Gaps** |
| --- | --- |
| Transmission risk due to inadequate spacing and layout | >1 patient in a single patient incubator/warmer |
| Transmission risk associated with the built environment | Lack of running water |
|  | Lack or inappropriate locations of HH stations (sinks) |
|  | Sink design which proliferates biofilm formation and creates bacterial reservoirs |
| Transmission risk due to contaminated environmental surfaces (e.g., table tops, counters, walls, floors) and linens (e.g., curtains, blankets) | Low compliance to proper cleaning and disinfection techniques/processes (e.g. lack of training, lack of supplies, lack of protocols, insufficient staffing) |
| Transmission due to contaminated medical equipment (e.g. shared equipment-portable x-rays, ultra sound, pulse ox, etc) | Low compliance to proper cleaning and disinfection techniques/processes (e.g. lack of training, lack of supplies, lack of protocols, insufficient staffing) |
| Transmission risk due to contaminated beds/incubators | Low compliance to proper cleaning and disinfection techniques/processes (e.g. lack of training, lack of supplies, lack of protocols, insufficient staffing) |
| Transmission risk due to contaminated semi-critical items (e.g. ventilator tubing, catheters, respiratory equipment) and critical items (e.g. scalpels) | Inadequate equipment to perform sterilization or reprocessing |
|  | Poor adherence to processing/sterilization standards |
|  | Reuse of single use items |
| Transmission risk due to contaminated humidifiers or suctions | Use of non-sterile water and lack of effective cleaning of reservoirs |
| Transmission risk due to contaminated HCW hands | Lack of supplies (soap, ABHR, paper, towels) |
|  | Low HCW compliance |
| Transmission risk due to contaminated medication preparation | Lack of or improper sterile compounding |
| Transmission risk due to unsafe injection practices | Improper multi-dose vial use (e.g., using same syringe for multiple uses, using a single dose vial for multiple doses)/ Reuse of single use needles/syringes |
|  | Improper sterile technique associated with administering IV medication |
| Transmission risk associated with central and peripheral lines | Lack of or improper aseptic technique for device insertion |
|  | Lack of adherence to central/peripheral line maintenance/care (e.g, daily assessments, dressing integrity, site cleanliness, prolonged use) |
|  | Non-adherence to steps/procedures for accessing central/peripheral line |
| Transmission risk due to inadequate clinical staffing and training | Inadequate nurse to neonate ratio |
|  | Poor patient safety culture |

ABHR: alcohol based hand rub, HCW: healthcare worker, HH: hand hygiene, IV: intravenous

**Appendix S5: Literature Review References**

1. Afjeh SA, Sabzehei MK, Karimi A, Shiva F, Shamshiri AR. Surveillance of ventilator-associated pneumonia in a neonatal intensive care unit: characteristics, risk factors, and outcome. Archives of Iranian Medicine. 2012;15(9):567-71.

2. Al Jarousha AM, El Qouqa IA, El Jadba AH, Al Afifi AS. An outbreak of Serratia marcescens septicaemia in neonatal intensive care unit in Gaza City, Palestine. Journal of Hospital Infection. 2008;70(2):119-26.

3. Anil M, Helvaci M, Ozkalay N, Toprak E, Anil AB, Dilek M, et al. Salmonella typhimurium outbreak in a neonatal unit in Turkey. Indian Journal of Pediatrics. 2009;76(6):629-33.

4. Antony B, Prasad BPMR. An outbreak of neonatal septicaemia by Enterobacter cloacae. Asian Pacific Journal of Tropical Disease. 2011;1(3):227-9.

5. Aragao PA, Oshiro IC, Manrique EI, Gomes CC, Matsuo LL, Leone C, et al. Pichia anomala outbreak in a nursery: exogenous source? Pediatric Infectious Disease Journal. 2001;20(9):843-8.

6. Azab SF, Sherbiny HS, Saleh SH, Elsaeed WF, Elshafiey MM, Siam AG, et al. Reducing ventilator-associated pneumonia in neonatal intensive care unit using "VAP prevention Bundle": a cohort study. BMC Infect Dis. 2015;15:314.

7. Babu MC, Tandur B, Sharma D, Murki S. Disposable diapers decrease the incidence of neonatal infections compared to cloth diapers in a level II neonatal intensive care unit. Journal of Tropical Pediatrics. 2015;61(4):250-4.

8. Bayramoglu G, Buruk K, Dinc U, Mutlu M, Yilmaz G, Aslan Y. Investigation of an outbreak of Serratia marcescens in a neonatal intensive care unit. Journal of Microbiology, Immunology & Infection. 2011;44(2):111-5.

9. Berger P, Barguellil F, Raoult D, Drancourt M. An outbreak of Halomonas phocaeensis sp. nov. bacteraemia in a neonatal intensive care unit. Journal of Hospital Infection. 2007;67(1):79-85.

10. Bouallegue O, Mzoughi R, Weill FX, Mahdhaoui N, Salem YB, Sboui H, et al. Outbreak of Pseudomonas putida bacteraemia in a neonatal intensive care unit. Journal of Hospital Infection. 2004;57(1):88-91.

11. Brito DV, Brito CS, Resende DS, Moreira do OJ, Abdallah VO, Gontijo Filho PP. Nosocomial infections in a Brazilian neonatal intensive care unit: a 4-year surveillance study. Revista Da Sociedade Brasileira de Medicina Tropical. 2010;43(6):633-7.

12. Brito DvDd, Oliveira EJ, Abdallah VOS, Darini ALdC, Gontijo Filho PP. An outbreak of Acinetobacter baumannii septicemia in a neonatal intensive care unit of a university hospital in Brazil. Brazilian Journal of Infectious Diseases. 2005;9(4):301-9.

13. Brito DvDd, Oliveira EJ, Darini ALdC, Abdallah VOS, Gontijo Filho PP. Nosocomial outbreaks due to Pseudomonas aeruginosa and Acinetobacter baumannii in a Neonatal Intensive Care Unit (NICU) of the Uberlandia Federal University Hospital. Brazilian Journal of Microbiology. 2003;34(Supp 1):27-8.

14. Brown SM, Lubimova AV, Khrustalyeva NM, Shulaeva SV, Tekhova I, Zueva LP, et al. Use of an alcohol-based hand rub and quality improvement interventions to improve hand hygiene in a Russian neonatal intensive care unit. Infect Control Hosp Epidemiol. 2003;24(3):172-9.

15. Buyukyavuz BI, Adiloglu AK, Onal S, Cubukcu SE, Cetin H. Finding the sources of septicemia at a neonatal intensive care unit: newborns and infants can be contaminated while being fed. Japanese Journal of Infectious Diseases. 2006;59(4):213-5.

16. Camargo LFA, Strabelli TMV, Ribeiro FG, Iwahashi ER, Ebaid M, Filho HHH, et al. Epidemiologic investigation of an outbreak of coagulase-negative Staphylococcus primary bacteremia in a newborn intensive care unit. Infection Control & Hospital Epidemiology. 1995;16(10):595-6.

17. Campos LC, Lobianco LF, Seki LM, Santos RMR, Asensi MD. Outbreak of Enterobacter hormaechei septicaemia in newborns caused by contaminated parenteral nutrition in Brazil. Journal of Hospital Infection. 2007;66(1):95.

18. Carrillo-Casas EM, Suastegui-Urquijo Z, Arroyo-Escalante S, Morales-Espinosa R, Moncada-Barron D, Hernandez-Delgado L, et al. E. coli outbreak in a neonate intensive care unit in a general hospital in Mexico City. Folia Microbiologica. 2013;58(3):229-34.

19. Chhapola V, Brar R. Impact of an educational intervention on hand hygiene compliance and infection rate in a developing country neonatal intensive care unit. International Journal of Nursing Practice. 2015;21(5):486-92.

20. Cleves D, Pino J, Patino JA, Rosso F, Velez JD, Perez P. Effect of chlorhexidine baths on central-line-associated bloodstream infections in a neonatal intensive care unit in a developing country. The Journal of hospital infection. 2018;100(3):e196-e9.

21. Couto RC, Pedrosa TM, Tofani Cde P, Pedroso ER. Risk factors for nosocomial infection in a neonatal intensive care unit. Infection Control & Hospital Epidemiology. 2006;27(6):571-5.

22. Dalben M, Varkulja G, Basso M, Krebs VLJ, Gibelli MA, van der Heijden I, et al. Investigation of an outbreak of Enterobacter cloacae in a neonatal unit and review of the literature. Journal of Hospital Infection. 2008;70(1):7-14.

23. Darmstadt GL, Badrawi N, Law PA, Ahmed S, Bashir M, Iskander I, et al. Topically applied sunflower seed oil prevents invasive bacterial infections in preterm infants in Egypt: a randomized, controlled clinical trial. Pediatric Infectious Disease Journal. 2004;23(8):719-25.

24. Darmstadt GL, Nawshad Uddin Ahmed AS, Saha SK, Azad Chowdhury MA, Alam MA, Khatun M, et al. Infection control practices reduce nosocomial infections and mortality in preterm infants in Bangladesh. Journal of Perinatology. 2005;25(5):331-5.

25. Darmstadt GL, Saha SK, Ahmed AS, Chowdhury MA, Law PA, Ahmed S, et al. Effect of topical treatment with skin barrier-enhancing emollients on nosocomial infections in preterm infants in Bangladesh: a randomised controlled trial. Lancet. 2005;365(9464):1039-45.

26. De Brito DVD, Von Dolinger EJO, Abdallah VOS, Darini ALC, Gontijo Filho PP. Two outbreaks of mixed etiology associated with central venous catheters inserted by phlebotomy in critical neonates. Brazilian Journal of Infectious Diseases. 2009;13(3):177-82.

27. de Souza Rugolo LM, Bentlin MR, Mussi-Pinhata M, de Almeida MF, Lopes JM, Marba ST, et al. Late-onset sepsis in very low birth weight infants: a Brazilian Neonatal Research Network Study. Journal of Tropical Pediatrics. 2014;60(6):415-21.

28. Deng C, Li X, Zou Y, Wang J, Wang J, Namba F, et al. Risk factors and pathogen profile of ventilator-associated pneumonia in a neonatal intensive care unit in China. Pediatrics International. 2011;53(3):332-7.

29. Dhaneria M, Jain S, Singh P, Mathur A, Lundborg CS, Pathak A. Incidence and Determinants of Health Care-Associated Blood Stream Infection at a Neonatal Intensive Care Unit in Ujjain, India: A Prospective Cohort Study. Diseases. 2018;6(1):30.

30. Djordjevic ZM, Markovic-Denic L, Folic MM, Igrutinovic Z, Jankovic SM. Health care-acquired infections in neonatal intensive care units: risk factors and etiology. American Journal of Infection Control. 2015;43(1):86-8.

31. Efird MM, Rojas MA, Lozano JM, Bose CL, Rojas MX, Rondon MA, et al. Epidemiology of nosocomial infections in selected neonatal intensive care units in Colombia, South America. Journal of Perinatology. 2005;25(8):531-6.

32. Ertugrul S, Aktar F, Yolbas I, Yilmaz A, Elbey B, Yildirim A, et al. Risk Factors for Health Care-Associated Bloodstream Infections in a Neonatal Intensive Care Unit. Iranian Journal of Pediatrics. 2016;26(5):e5213.

33. Fotedar R, Banerjee U, Chaudhary AR. Outbreak of systemic candidiasis in low birth weight preterm infants at a neonatal intensive care unit. Journal de Mycologie Medicale. 2000;10(2):100-4.

34. Freitas BA, Peloso M, Manella LD, Franceschini Sdo C, Longo GZ, Gomes AP, et al. Late-onset sepsis in preterm children in a neonatal intensive care unit: a three-year analysis. Revista Brasileira de Terapia Intensiva. 2012;24(1):79-85.

35. Gadallah MA, Aboul Fotouh AM, Habil IS, Imam SS, Wassef G. Surveillance of health care-associated infections in a tertiary hospital neonatal intensive care unit in Egypt: 1-year follow-up. American Journal of Infection Control. 2014;42(11):1207-11.

36. Ganeswire R, Thong KL, Puthucheary SD. Nosocomial outbreak of Enterobacter gergoviae bacteraemia in a neonatal intensive care unit. Journal of Hospital Infection. 2003;53(4):292-6.

37. Gathwala G, Sharma D, Bhakhri B. Effect of topical application of chlorhexidine for umbilical cord care in comparison with conventional dry cord care on the risk of neonatal sepsis: a randomized controlled trial. Journal of Tropical Pediatrics. 2013;59(3):209-13.

38. Ghadage D, Bal A. Outbreak of neonatal meningitis caused by Salmonella enterica serotype Worthington. Indian Journal of Pathology and Microbiology. 2003;46(2):268-70.

39. Gill CJ, Mantaring JB, Macleod WB, Mendoza M, Mendoza S, Huskins WC, et al. Impact of enhanced infection control at 2 neonatal intensive care units in the Philippines. Clinical Infectious Diseases. 2009;48(1):13-21.

40. Gray J, Arvelo W, McCracken J, Lopez B, Lessa FC, Kitchel B, et al. An outbreak of Klebsiella pneumoniae late-onset sepsis in a neonatal intensive care unit in Guatemala. American Journal of Infection Control. 2012;40(6):516-20.

41. Guducuoglu H, Gultepe B, Otlu B, Bektas A, Yildirim O, Tuncer O, et al. Candida albicans outbreak associated with total parenteral nutrition in the neonatal unit. Indian Journal of Medical Microbiology. 2016;34(2):202-7.

42. Gungor S, Ozen M, Akinci A, Durmaz R. A Chryseobacterium Meningosepticum outbreak in a neonatal ward. Infection Control and Hospital Epidemiology. 2003;24(8):613-7.

43. Habsah H, Zeehaida M, Van Rostenberghe H, Noraida R, Wan Pauzi WI, Fatimah I, et al. An outbreak of Pantoea spp. in a neonatal intensive care unit secondary to contaminated parenteral nutrition. Journal of Hospital Infection. 2005;61(3):213-8.

44. Halim MMA, Eyada IK, Tongun RM. Prevalence of multidrug drug resistant organisms and hand hygiene compliance in surgical NICU in Cairo University Specialized Pediatric Hospital. Egyptian Pediatric Association Gazette. 2018;66(4):103-11.

45. Hammami A, Arlet G, Ben Redjeb S. Nosocomial outbreak of acute gastroenteritis in a neonatal intensive care unit in Tunisia caused by multiply drug resistant Salmonella wien producing SHV-2 beta-lactamase. European Journal of Clinical Microbiology and Infectious Diseases. 1991;10(8):641-6.

46. Hernandez-Castro R, Arroyo-Escalante S, Carrillo-Casas EM, Moncada-Barron D, Alvarez-Verona E, Hernandez-Delgado L, et al. Outbreak of Candida parapsilosis in a neonatal intensive care unit: a health care workers source. European Journal of Pediatrics. 2010;169(7):783-7.

47. Hosoglu S, Hascuhadar M, Yasar E, Uslu S, Aldudak B. Control of an Acientobacter baumannii outbreak in a neonatal ICU without suspension of service: a devastating outbreak in Diyarbakir, Turkey. Infection. 2012;40(1):11-8.

48. Hu HB, Huang HJ, Peng QY, Lu J, Lei XY. Prospective study of colonization and infection because of Pseudomonas aeruginosa in mechanically ventilated patients at a neonatal intensive care unit in China. American Journal of Infection Control. 2010;38(9):746-50.

49. Huang Y, Zhuang S, Du M. Risk factors of nosocomial infection with extended-spectrum beta-lactamase-producing bacteria in a neonatal intensive care unit in China. Infection. 2007;35(5):339-45.

50. Indarso F, Harianto A, Nada A, Aly H. Outbreak of neonatal cellulites and septicemia caused by Salmonella worthington. Journal of Pediatric Infectious Diseases. 2008;3(4):241-4.

51. Issack MI, Neetoo Y. An outbreak of Elizabethkingia meningoseptica neonatal meningitis in mauritius. Journal of Infection in Developing Countries. 2011;5(12):834-9.

52. Jain S, Gaind R, Kothari C, Sehgal R, Shamweel A, Thukral SS, et al. VEB-1 extended-spectrum beta-lactamase-producing multidrug-resistant Proteus mirabilis sepsis outbreak in a neonatal intensive care unit in India: clinical and diagnostic implications. JMM Case Reports. 2016;3(4):e005056.

53. Kasim K, El-Sadak AA, Zayed K, Abdel-Wahed A, Mosaad M. Nosocomial infections in a neonatal intensive care unit. Middle East Journal of Scientific Research. 2014;19(1):1-7.

54. Kawagoe JY, Segre CA, Pereira CR, Cardoso MF, Silva CV, Fukushima JT. Risk factors for nosocomial infections in critically ill newborns: a 5-year prospective cohort study. American Journal of Infection Control. 2001;29(2):109-14.

55. Khan MA, Abdur-Rab M, Israr N, Ilyas M, Ahmad F, Kundi Z, et al. Transmission of Salmonella worthington by oropharyngeal suction in hospital neonatal unit. Pediatric Infectious Disease Journal. 1991;10(9):668-72.

56. Khurana S, Saini SS, Sundaram V, Dutta S, Kumar P. Reducing Healthcare-associated Infections in Neonates by Standardizing and Improving Compliance to Aseptic Non-touch Techniques: A Quality Improvement Approach. Indian Pediatrics. 2018;55(9):748-52.

57. Krajcinovic SS, Doronjski A, Barisic N, Stojanovic V. Risk Factors for Neonatal Sepsis and Method for Reduction of Blood Culture Contamination. Malawi Medical Journal. 2015;27(1):20-4.

58. Kuboyama RH, de Oliveira HB, Moretti-Branchini ML. Molecular epidemiology of systemic infection caused by Enterobacter cloacae in a high-risk neonatal intensive care unit. Infection Control & Hospital Epidemiology. 2003;24(7):490-4.

59. Landre-Peigne C, Ka AS, Peigne V, Bougere J, Seye MN, Imbert P. Efficacy of an infection control programme in reducing nosocomial bloodstream infections in a Senegalese neonatal unit. Journal of Hospital Infection. 2011;79(2):161-5.

60. Lee JK. Two outbreaks of Burkholderia cepacia nosocomial infection in a neonatal intensive care unit. Journal of Paediatrics & Child Health. 2008;44(1-2):62-6.

61. Li Z, Xiao Z, Li Z, Zhong Q, Zhang Y, Xu F. 116 cases of neonatal early-onset or late-onset sepsis: A single center retrospective analysis on pathogenic bacteria species distribution and antimicrobial susceptibility. International journal of clinical and experimental medicine. 2013;6(8):693-9.

62. Lin R, Wu B, Xu XF, Liu XC, Ye H, Ye GY. Extended-spectrum beta-lactamase-producing Klebsiella pneumoniae infection in a neonatal intensive care unit. World Journal of Pediatrics. 2012;8(3):268-71.

63. Macias AE, Munoz JM, Galvan A, Gonzalez JA, Medina H, Alpuche C, et al. Nosocomial bacteremia in neonates related to poor standards of care. Pediatric Infectious Disease Journal. 2005;24(8):713-6.

64. Mahajan R, Mathur M, Kumar A, Gupta P, Faridi MM, Talwar V. Nosocomial outbreak of Salmonella typhimurium infection in a nursery intensive care unit (NICU) and paediatric ward. The Journal of communicable diseases. 1995;27(1):10-4.

65. Marofi M, Bijani N, Abdeyazdan Z, Barekatain B. The Impact of an Educational Program Regarding Total Parenteral Nutrition on Infection Indicators in Neonates Admitted to the Neonatal Intensive Care Unit. Iranian Journal of Nursing and Midwifery Research. 2017;22(6):486-9.

66. Martinez-Aguilar G, Alpuche-Aranda CM, Anaya C, Alcantar-Curiel D, Gayosso C, Daza C, et al. Outbreak of nosocomial sepsis and pneumonia in a newborn intensive care unit by multiresistant extended-spectrum beta-lactamase-producing Klebsiella pneumoniae: High impact on mortality. Infection Control and Hospital Epidemiology. 2001;22(11):725-8.

67. Medeiros AFdV, Herdy Alves V, Sobrinho Valete CO, Dórea Paiva E, Pereira Rodrigues D. The correlation between invasive care procedures and the occurrence of neonatal sepsis. Acta Paulista de Enfermagem. 2016;29(5):573-8.

68. Miranda-Novales G, Leanos-Miranda B, Diaz-Ramos R, Gonzalez-Tejeda L, Peregrino-Bejarano L, Villegas-Silva R, et al. An outbreak due to Serratia marcescens in a neonatal intensive care unit typed by 2-day pulsed field gel electrophoresis protocol. Archives of Medical Research. 2003;34(3):237-41.

69. Mittal N, Nair D, Gupta N, Rawat D, Kabra S, Kumar S, et al. Outbreak of Acinetobacter spp septicemia in a neonatal ICU. Southeast Asian Journal of Tropical Medicine & Public Health. 2003;34(2):365-6.

70. Moehario LH, Tjoa E, Rohsiswatmo R, Nursyirwan SR. Microbes profile from blood stream infection cases and their relationship to those of environment in Neonatal unit. Medical Journal of Indonesia. 2012;21(1):32-7.

71. Mohamed SS, Ali BAEM, Abd-El-Karim HE, Masoed ES. Nurses' knowledge and performance regarding infection control during milk formula preparation and its effect on neonates at El-Minia city hospitals. The Journal of American Science. 2012;8(10):782-7.

72. Mohammed D, El Seifi OS. Bacterial nosocomial infections in neonatal intensive care unit, Zagazig University Hospital, Egypt. Egyptian Pediatric Association Gazette. 2014;62(3-4):72-9.

73. Moodley P, Coovadia YM, Sturm AW. Intravenous glucose preparation as the source of an outbreak of extended-spectrum beta-lactamase-producing Klebsiella pneumoniae infections in the neonatal unit of a regional hospital in KwaZulu-Natal. South African Medical Journal Suid-Afrikaanse Tydskrif Vir Geneeskunde. 2005;95(11):861-4.

74. Moore KL, Kainer MA, Badrawi N, Afifi S, Wasfy M, Bashir M, et al. Neonatal sepsis in Egypt associated with bacterial contamination of glucose-containing intravenous fluids. Pediatric Infectious Disease Journal. 2005;24(7):590-4.

75. Mshana SE, Gerwing L, Minde M, Hain T, Domann E, Lyamuya E, et al. Outbreak of a novel Enterobacter sp. carrying blaCTX-M-15 in a neonatal unit of a tertiary care hospital in Tanzania. International Journal of Antimicrobial Agents. 2011;38(3):265-9.

76. Muley VA, Pol SS, Dohe VB, Nagdawane RP, Arjunwadkar VP, Pandit DP, et al. Neonatal outbreak of Salmonella worthington in a general hospital. Indian Journal of Medical Microbiology. 2004;22(1):51-3.

77. Nagarathnamma T, Chunchanur SK, Rudramurthy SM, Vineetha KR, Karthik R, Jillwin J, et al. Outbreak of Pichia kudriavzevii fungaemia in a neonatal intensive care unit. Journal of Medical Microbiology. 2017;66(12):1759-64.

78. Nagata E, Brito AS, Matsuo T. Nosocomial infections in a neonatal intensive care unit: incidence and risk factors. American Journal of Infection Control. 2002;30(1):26-31.

79. Narayan SA, Kool JL, Vakololoma M, Steer AC, Mejia A, Drake A, et al. Investigation and control of an outbreak of Enterobacter aerogenes bloodstream infection in a neonatal intensive care unit in Fiji. Infection Control & Hospital Epidemiology. 2009;30(8):797-800.

80. Newman MJ. Multiple-resistant Salmonella group G outbreak in a neonatal intensive care unit. West African Journal of Medicine. 1996;15(3):165-9.

81. Ng PC, Wong HL, Lyon DJ, So KW, Liu F, Lam RKY, et al. Combined use of alcohol hand rub and gloves reduces the incidence of late onset infection in very low birthweight infants. Archives of Disease in Childhood. 2004;89(4):F336-F40.

82. Oliveira Ad, Sanches P, Lyra JC, Bentlin MR, Rugolo LMSS, Cunha MdLRdSd. Risk factors for infection with coagulase-negative staphylococci in newborns from the Neonatal Unit of a Brazilian University Hospital. Clinical Medicine Insights: Pediatrics. 2012;6:1-9.

83. Pessoa-Silva CL, Meurer Moreira B, Camara Almeida V, Flannery B, Almeida Lins MC, Mello Sampaio JL, et al. Extended-spectrum beta-lactamase-producing Klebsiella pneumoniae in a neonatal intensive care unit: risk factors for infection and colonization. Journal of Hospital Infection. 2003;53(3):198-206.

84. Picheansathian W, Pearson A, Suchaxaya P. The effectiveness of a promotion programme on hand hygiene compliance and nosocomial infections in a neonatal intensive care unit. International Journal of Nursing Practice. 2008;14(4):315-21.

85. Qadir M, Qamar FN, Resham S, Ali R, Khalil A, Ahmed S, et al. Effectiveness of simple strategies in reducing multidrug resistant blood stream infections in Neonatal Intensive Care Unit of tertiary care hospital in Karachi, Pakistan. JPMA - Journal of the Pakistan Medical Association. 2015;65(1):72-5.

86. Rameshwarnath S, Naidoo S. Risk factors associated with nosocomial infections in the Neonatal Intensive Care Unit at Mahatma Gandhi Memorial hospital between 2014 and 2015. Southern African Journal of Infectious Diseases. 2018;33(4):93-100.

87. Rangel UV, Gomes Junior SC, Costa AM, Moreira ME. Variables associated with peripherally inserted central catheter related infection in high risk newborn infants. Revista Latino-Americana de Enfermagem. 2014;22(5):842-7.

88. Rastogi V, Nirwan PS, Jain S, Kapil A. Nosocomial outbreak of septicaemia in neonatal intensive care unit due to extended spectrum beta-lactamase producing Klebsiella pneumoniae showing multiple mechanisms of drug resistance. Indian J Med Microbiol. 2010;28(4):380-4.

89. Resende DS, O JM, Brito D, Abdallah VO, Gontijo Filho PP. Reduction of catheter-associated bloodstream infections through procedures in newborn babies admitted in a university hospital intensive care unit in Brazil. Revista Da Sociedade Brasileira de Medicina Tropical. 2011;44(6):731-4.

90. Resende DS, Peppe AL, dos Reis H, Abdallah VO, Ribas RM, Gontijo Filho PP. Late onset sepsis in newborn babies: epidemiology and effect of a bundle to prevent central line associated bloodstream infections in the neonatal intensive care unit. The Brazilian journal of infectious diseases : an official publication of the Brazilian Society of Infectious Diseases. 2015;19(1):52-7.

91. Rohsiswatmo R, Rafika S, Marsubrin PM. Prevention and control of blood stream infection using the balanced scorecard approach. Acta Med Indones. 2014;46(3):209-16.

92. Rojas MA, Efird MM, Lozano JM, Bose CL, Rojas MX, Rondon MA, et al. Risk factors for nosocomial infections in selected neonatal intensive care units in Colombia, South America. Journal of Perinatology. 2005;25(8):537-41.

93. Romanelli RM, Anchieta LM, Carvalho EA, Gloria e Silva LF, Nunes RV, Mourao PH, et al. Risk factors for laboratory-confirmed bloodstream infection in neonates undergoing surgical procedures. Brazilian Journal of Infectious Diseases. 2014;18(4):400-5.

94. Romanelli RM, Anchieta LM, Mourao MV, Campos FA, Loyola FC, Mourao PH, et al. Risk factors and lethality of laboratory-confirmed bloodstream infection caused by non-skin contaminant pathogens in neonates. Jornal de Pediatria. 2013;89(2):189-96.

95. Rosenthal VD, Duenas L, Sobreyra-Oropeza M, Ammar K, Navoa-Ng JA, de Casares AC, et al. Findings of the International Nosocomial Infection Control Consortium (INICC), part III: effectiveness of a multidimensional infection control approach to reduce central line-associated bloodstream infections in the neonatal intensive care units of 4 developing countries. Infection Control & Hospital Epidemiology. 2013;34(3):229-37.

96. Rosenthal VD, Rodriguez-Calderon ME, Rodriguez-Ferrer M, Singhal T, Pawar M, Sobreyra-Oropeza M, et al. Findings of the International Nosocomial Infection Control Consortium (INICC), Part II: Impact of a multidimensional strategy to reduce ventilator-associated pneumonia in neonatal intensive care units in 10 developing countries. Infection Control & Hospital Epidemiology. 2012;33(7):704-10.

97. Rundjan L, Rohsiswatmo R, Paramita TN, Oeswadi CA. Closed catheter access system implementation in reducing the bloodstream infection rate in low birth weight preterm infants. Frontiers in pediatrics. 2015;3:20.

98. Sadeghi-Moghaddam P, Arjmandnia M, Shokrollahi M, Aghaali M. Does training improve compliance with hand hygiene and decrease infections in the neonatal intensive care unit? A prospective study. J Neonatal Perinatal Med. 2015;8(3):221-5.

99. Salam RA, Darmstadt GL, Bhutta ZA. Effect of emollient therapy on clinical outcomes in preterm neonates in Pakistan: a randomised controlled trial. Archives of Disease in Childhood Fetal & Neonatal Edition. 2015;100(3):F210-5.

100. Saritha K. Outbreak of MRSA in the Neonatal Intensive Care Unit of a tertiary care hospital - Transmission from nursing personnel. Journal of Clinical and Diagnostic Research. 2009;3(3):1510-2.

101. Shalini S, Ashwini H, Saldanha DRM, Saritha K, Arvind N. An outbreak of extended spectrum beta -lactamase producing Klebsiella pneumoniae in a neonatal intensive care unit. Indian Journal of Pathology & Microbiology. 2007;50(3):669-70.

102. Sultan AM, Seliem WA. Identifying Risk Factors for Healthcare-Associated Infections Caused by Carbapenem-Resistant Acinetobacter baumannii in a Neonatal Intensive Care Unit. Sultan Qaboos University Medical Journal. 2018;18(1):e75-e80.

103. Sumer S, Turk Dagi H, Findik D, Arslan U, Aktug Demir N, Ural O, et al. Two outbreaks of ESBL-producing Klebsiella pneumoniae in a neonatal intensive care unit. Pediatrics international : official journal of the Japan Pediatric Society. 2014;56(2):222-6.

104. Taneja N, Das A, Raman Rao DSV, Jain N, Singh M, Sharma M. Nosocomial outbreak of diarrhoea by enterotoxigenic Escherichia coli among preterm neonates in a tertiary care hospital in India: Pitfalls in healthcare. Journal of Hospital Infection. 2003;53(3):193-7.

105. Tapia-Rombo CA, Ugarte-Torres RG, Alvarez-Vazquez E, Salazar-Acuna AH. Risk factors for intrahospital infection in newborns. Archives of Medical Research. 2001;32(4):304-11.

106. TaTavora AC, Castro AB, Militao MA, Girao JE, Ribeiro Kde C, Tavora LG. Risk factors for nosocomial infection in a Brazilian neonatal intensive care unit. Brazilian Journal of Infectious Diseases. 2008;12(1):75-9.

107. Touati A, Achour W, Cherif A, Hmida HB, Afif FB, Jabnoun S, et al. Outbreak of Acinetobacter baumannii in a neonatal intensive care unit: antimicrobial susceptibility and genotyping analysis. Annals of Epidemiology. 2009;19(6):372-8.

108. Von Dolinger Brito D, Matos C, Abdalla VV, Filho DA, Pinto Gontijo PF. An Outbreak of Nosocomial Infection Caused by ESBLs Producing Serratia marcescens in a Brazilian Neonatal Unit. Brazilian Journal of Infectious Diseases. 1999;3(4):149-55.

109. Wadile R, Bhate V. Study of clinical spectrum and risk factors of neonatal candidemia. Indian Journal of Pathology and Microbiology. 2015;58(4):472-4.

110. Yapicioglu H, Gokmen TG, Yildizdas D, Koksal F, Ozlu F, Kale-Cekinmez E, et al. Pseudomonas aeruginosa infections due to electronic faucets in a neonatal intensive care unit. Journal of Paediatrics and Child Health. 2012;48(5):430-4.

111. Yu Y, Du L, Yuan T, Zheng J, Chen A, Chen L, et al. Risk factors and clinical analysis for invasive fungal infection in neonatal intensive care unit patients. American Journal of Perinatology. 2013;30(7):589-94.

112. Zhou Q, Lee SK, Hu XJ, Jiang SY, Chen C, Wang CQ, et al. Successful reduction in central line-associated bloodstream infections in a Chinese neonatal intensive care unit. American Journal of Infection Control. 2015;43(3):275-9.

113. Zhou Q, Lee SK, Jiang SY, Chen C, Kamaluddeen M, Hu XJ, et al. Efficacy of an infection control program in reducing ventilator-associated pneumonia in a Chinese neonatal intensive care unit. American Journal of Infection Control. 2013;41(11):1059-64.

**Table S1: Intervention measures (n=27)**

| **Interventions** | **N** | **(%)** |
| --- | --- | --- |
| Multimodal strategy | 8 | 30 |
| - Hand hygiene | *7* |  |
| - Isolation/cohorting | *3* |  |
| - CHG bathing | *2* |  |
| - HCW training/education | *2* |  |
| - PPE use | *2* |  |
| - Environmental cleaning | *2* |  |
| Hand hygiene | 5 | 18 |
| Bundle | 5 | 18 |
| Injection safety | 1 | 4 |
| Other | 8 | 30 |
| **Total** | **27** | **100** |
